# Intelligent Positioning for a Commercial Mobile Platform in Seamless Indoor/Outdoor Scenes based on Multi-sensor Fusion

**DOI:** 10.3390/s19071696

**Published:** 2019-04-09

**Authors:** Dongsheng Wang, Yongjie Lu, Lei Zhang, Guoping Jiang

**Affiliations:** 1 Jiangsu Engineering Lab for IOT Intelligent Robots, Nanjing 210023, China; luyongjie2016@126.com (Y.L.); jianggp@njupt.edu.cn (G.J.); 2 School of Automation, Nanjing University of Posts and Telecommunications, Nanjing 210023, China; 3 School of IOT, Nanjing University of Posts and Telecommunications, Nanjing 210023, China; lei.z@njupt.edu.cn

**Keywords:** magnetometer, heading angle, integrated positioning, Kalman filter, embedded system

## Abstract

Many traffic occasions such as tunnels, subway stations and underground parking require accurate and continuous positioning. Navigation and timing services offered by the Global Navigation Satellite System (GNSS) is the most popular outdoor positioning method, but its signals are vulnerable to interference, leading to a degraded performance or even unavailability. The combination of magnetometer and Inertial Measurement Unit (IMU) is one of the commonly used indoor positioning methods. Within the proposed mobile platform for positioning in seamless indoor and outdoor scenes, the data of magnetometer and IMU are used to update the positioning when the GNSS signals are weak. Because the magnetometer is susceptible to environmental interference, an intelligent method for calculating heading angle by magnetometer is proposed, which can dynamically calculate and correct the heading angle of the mobile platform in a working environment. The results show that the proposed method of calculating heading angle by magnetometer achieved better performance with interference existence. Compared with the uncorrected heading angle, the corrected accuracy results could be improved by 60%, and the effect was more obvious when the interference was stronger. The error of overall positioning trajectory and true trajectory was within 2 m.

## 1. Introduction

Providing pedestrians and vehicles with continuous positioning is a challenging but important topic [1,2,3]. In infrastructure-free navigation, it is necessary for mobile platform to provide accurate positioning in seamless indoor/outdoor occasions based on all the required sensors [4,5,6].

In recent years, various real-time applications benefit from services provided by localization systems due to the advent of new sensing and communication technologies [7]. The Global Navigation Satellite System (GNSS) provides sufficiently accurate information including time, position and velocity [8]. However, GNSS cannot work when an unobstructed sight line to four or more GNSS satellites is lacking. In environments such as urban canyons and tunnels, it is difficult for GNSS to provide continuous and reliable positioning [9,10]. Since the GNSS enables localization only in outside scene, indoor positioning and navigation applications have to use alternative technologies [11], such as Wi-Fi, Bluetooth, Inertial Measurement Units (IMU) and magnetometer. However, each of the techniques has its own advantages and disadvantages. For example, iBeacon technology, which relies on the Bluetooth Low Energy (BLE) standard to create stationary constellations of low-power beacons, can be used to determine the indoor position of mobile terminals or signaling points of interest [12]. However, it needs to establish a base station in advance, and thus it is not suitable for all occasions, such as tunnels, underground garages, etc. Wi-Fi positioning technology also needs to establish a base station, so it is not considered in this article. Therefore, these technical solutions are not suitable for the platform. Positioning techniques based on IMU estimate the step size and motion direction with its information [13,14]. This technique is widely applied, as it does not depend on external base stations or signal features. It has accurate positioning within a short distance, but its accuracy will sharply deteriorate by the cumulative error over time [15]. Positioning techniques based on magnetic fields is achieved using the distribution difference of the magnetic field at different locations on the Earth. The navigation system measures the magnetic field information of the space, including the geomagnetic field information and the interference magnetic field information. Therefore, magnetometers have some problems in the compensation of magnetic field. Especially in the case of constant changes in the external magnetic field environment, the magnetometer has great difficulty in error correction and compensation [16].

As the signal of GNSS satellites broadcasts in the civil field, it includes the high frequency random oscillating interference signal. Thus, all derived satellite signals will generate high frequency oscillation [17]. To improve the positioning accuracy, it is necessary to filter the GNSS observation signal to the position of the carrier. An effective solution is to integrate the GNSS receiver with the Inertial Navigation System (INS) owing to their complementary feature. The Kalman filter and its extension methods are widely employed to integrate the GNSS and INS to balance the performance of the two systems. Typically, the output of INS is used as a prediction value, and the output of GNSS as a measurement value. The Kalman filter estimates the new state recursively based on the state parameter at the previous moment and the observed value at the current moment. It is in the order of “prediction–measurement–correction”. According to the measured values of the system, random interference would be eliminated, and the state of the system is reproduced [18]. 

When the carrier enters a place with weak GNSS signals, it requires other tools for replacement. Since the INS can only provide short-term accuracy, it is not suitable for long-term indoor positioning. Therefore, it is necessary to choose a new way. The combination of Global Positioning System and magnetometers is used in the platform in [19]. According to the position value of the previous point, the current position value is calculated by the acceleration and direction provided by the accelerometer and magnetometer, respectively. However, the magnetic field strength measured by the magnetometers is easily disturbed by the surrounding environment, and the measurement error leads to an inaccurate heading angle [20]. This problem is not considered in [19]. At present, the method of correcting and compensating the magnetometer in the navigation system is very common, and some methods are mature, which can meet certain precision requirements [21,22]. However, most methods only consider the correction and compensation of the navigation system when the external environment remains unchanged. Therefore, these methods are not suitable for dealing with changes in the external magnetic field environment. Magnetic fingerprinting based indoor Localization (MaLoc) is an indoor positioning system, which is based on ambient magnetic field measurements using smartphones [23]. The system consists of a client running on smartphones and a server. The client collects magnetic and inertial sensor data and performs step counting, heading change between two contiguous steps, and accesses magnetic values in each step. The system has shown an average accuracy of the order of 1–2 m in a large building. 

In this paper, we aim at designing a mobile platform that enables positioning in seamless indoor/outdoor scenes. The trajectory estimation method is adopted in the indoor positioning. The heading angle and the acceleration values are provided by the magnetometer and the accelerometer, respectively. Based on the distribution characteristics of the geomagnetic field and the heading angle calculation principle, this paper proposes a dynamic calculation and correction method for the heading angle of the magnetometer navigation system. First, a dynamic error model of the magnetometer is established. Then, the correspondences of the magnetometer triaxial output at two adjacent moments are analyzed, and the formula containing the heading angle information is obtained. Then, according to the distribution characteristics of the magnetic field at the two adjacent moments, an additional formula for solving the heading angle is obtained. Finally, the heading angle can be solved by combining the above formulas. This method effectively realizes the correction and compensation of the magnetometer and improves the accuracy of the navigation system. In addition, the outdoor positioning adopts the method, which uses GNSS and INS data in combination with Kalman filtering. Further, according to the design requirements, it needs to meet the control and calculation capabilities of the entire system. Therefore, the processor unit adopts a dual-core architecture chip, which is combined by DSP kernel and ARM kernel. The Linux operation runs on ARM kernel and is responsible for the control of the entire system and data acquisition. DSP kernel is used for the data fusion navigation algorithm. The method of magnetometer calibration proposed in our paper has an accuracy within 2 m in indoor positioning. Compared with the system in [23], our system cost is also greatly reduced. The novelty of this paper is the method of dynamically solving the heading angle based on magnetometer.

The remainder of this paper is organized as follows. In Section 2, the positioning solution and location update principle are described. The methods of calculating the yaw angle and GNSS filtering are described in Section 3. The experimental measurement and performance test results are shown in Section 4. Finally, the conclusions are given in Section 5.

## 2. Related Work

### 2.1. Positioning Solution

**Figure 1:**
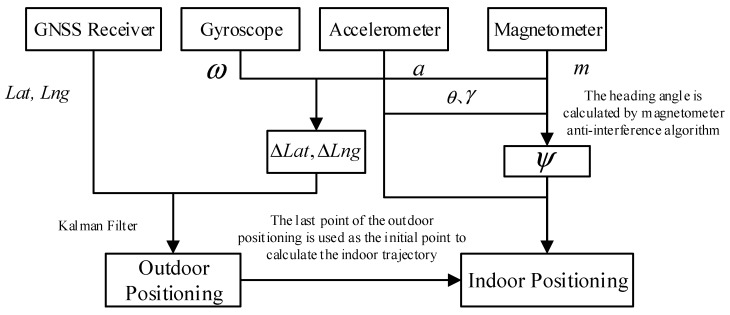
Positioning solution of the mobile positioning platform.

The mobile positioning platforms shown in Figure 1 are divided into outdoor and indoor occasions. GNSS is used in outdoor part, and the trajectory estimation method is adopted in indoor part. Since the noise in the GNSS signal causes the inaccurate positioning, we combine the angular velocity and the acceleration to calculate the increment, and then correct the outdoor positioning value with Kalman filtering. When the carrier enters the room, the last point of the outdoor positioning is used as the initial point to calculate the indoor trajectory. The magnetometer is used to provide the direction of the trajectory estimation. Since the magnetometer is susceptible to the surrounding magnetic field, we introduce a magnetometer anti-interference algorithm to dynamically calculate the heading angle.

### 2.2. Positioning Update

**Figure 2:**
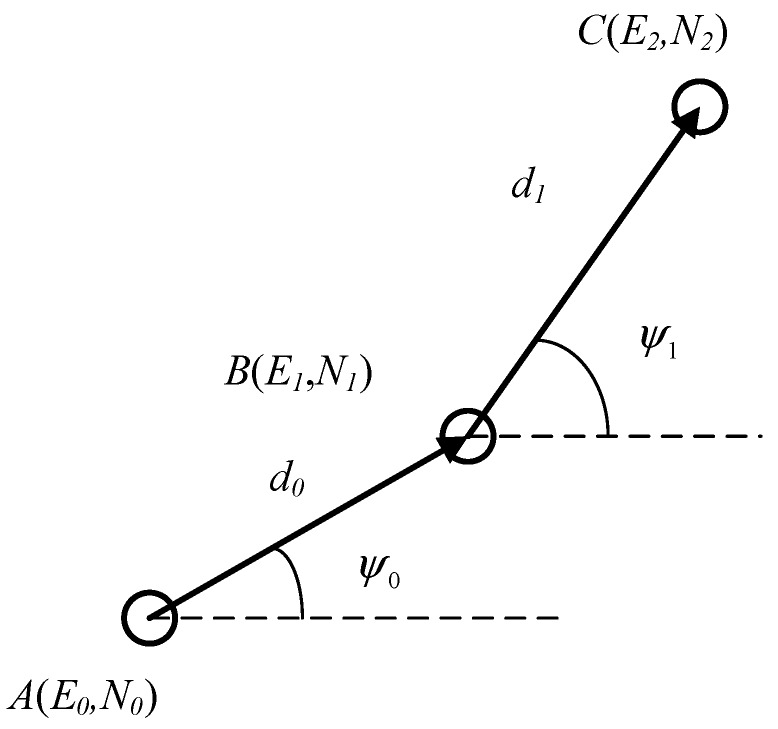
Track positioning.

The data of magnetometer and INS are used to update the positioning when the GNSS signals are weak. As shown in Figure 2, to update the coordinates of the next moment, it is necessary to know the coordinate value $N_{n}$ and $E_{n}$ of the last moment, the distance d of the motion, and the heading angle ψ. The formula is described as follows:

(1) $$\{\begin{array}{l} E_{n}=E_{0}+\sum_{i=0}^{n-1}d_{i}\cdot cos\psi_{i}\\ N_{n}=N_{0}+\sum_{i=0}^{n-1}d_{i}\cdot sin\psi_{i} \end{array}$$

**Figure 3:**
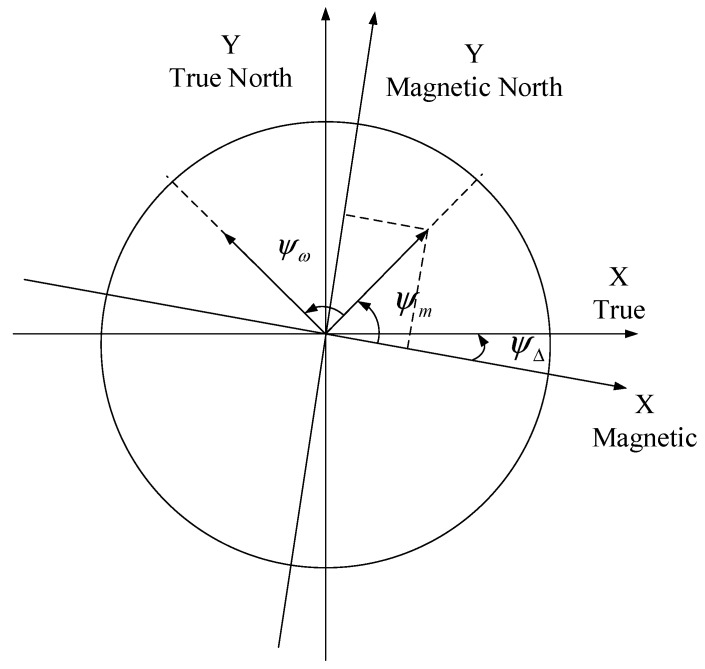
Heading angle conversion

Figure 2 is the grid coordinates, which need to establish a unified relationship with the latitude and longitude, as shown in Figure 3. We made the IMU and the magnetometer have the same three-axis direction. Then, the heading angle ψ in Equation (1) is composed of these three parameters: the initial direction $\psi_{m}$ provided by the magnetometer, the gyroscope provides a rotation angle $\psi_{\omega }$, and the angle difference $\psi_{\Delta }$ between magnetic north and true north. This variable $\psi_{\Delta }$ takes a value based on different geographic locations. In this article, the value is a negative number. The coordinates of the latitude and longitude are updated, as shown by Equations (2) and (3).

(2) $$Lat\left(k+1\right)=Lat\left(k\right)+\left[v\left(k\right)T_{0}+\frac{1}{2}T_{0}^{2}a\left(k\right)\right]sin(\psi_{\omega }+\psi_{m}+\psi_{\Delta })A$$

(3) $$Lng\left(k+1\right)=Lng\left(k\right)+\left[v\left(k\right)T_{0}+\frac{1}{2}T_{0}^{2}a\left(k\right)\right]cos(\psi_{\omega }+\psi_{m}+\psi_{\Delta })B$$

where $T_{0}$ is set to be the sampling time, Lat(k) is set to be the latitude of carrier at the $kT_{0}$ time, and Lng(k) is set to be the longitude of carrier at the $kT_{0}$ time. v(k) denotes the speed of the carrier in the moving direction. a(k) is the acceleration of the carrier. A and B indicate the proportion of length in meters to latitude and longitude locally.

## 3. Correction Methods Involved in the Platform

### 3.1. Calculation of Yaw Angle

The method of dynamically solving the heading angle based on the magnetometer is proposed in this paper, and is mainly divided into the following steps. First, the source of error in magnetometer measurements are analyzed and an error model for the magnetometer is built accordingly. The correspondence between the three-axis output of the magnetometers in the two adjacent moments is compared, and then is combined with the distribution characteristics of the geomagnetic field. A set of formulas containing the information of the heading angle is obtained. Finally, the heading angle can be calculated by solving the formulas.

The errors of magnetometers can be divided into two categories, one is the instrument error existing in the magnetometer itself, and the other is the error caused by the external environment. The first type of error can be considered as not changing with the external environment. It can be obtained by one correction. When the external environment changes, the second type of error will change accordingly, thus it can be set as a variable parameter. Therefore, the general error model of the magnetometer can be composed of the two types of errors as follows:

(4) $${\hat{M}}^{h}=\bar{N}M^{b}+m^{b}+E+E_{0}$$

$M^{h}$ is the triaxial output vector of the magnetometer. $\bar{N}$ is the total error matrix. $M^{b}$ is the local magnetic field vector. $m^{b}$ is the zero offset error vector. E is the error vector caused by the external environment. $E_{0}$ is the measurement error vector, which is generally considered to be white Gaussian noise, and can be ignored.

Regardless of the white Gaussian noise, the error model of the magnetometer can be obtained through a series of simplification changes as follows:

(5) $${\hat{M}}^{b}={\bar{N}}^{-1}\left({\hat{M}}^{h}-m^{b}-E\right)$$

${\hat{M}}^{b}$ is the triaxial output vector of the corrected magnetometer. $N^{-1}$ is the inverse matrix of N.

Let $M^{h}={\hat{M}}^{h}-m^{b}$, $N={\bar{N}}^{-1}$, then the above formula is expressed as:

(6) $${\hat{M}}^{b}=N\left(M^{h}-E\right)$$

In the above formula, the total error matrix N and the zero offset error vector $m^{b}$ do not change with the external environment. $m^{b}$ can be corrected once by the existing compensation method. The error vector E caused by the external magnetic field environment is an unknown variable.

When the magnetometer is in the horizontal position, the vertical direction does not need to be considered; only the data of the X and Y axes are used to calculate the heading angle. However, the magnetometer does not always maintain a horizontal position during the movement, and it would be a slight tilt, which means the magnetic field strength measured on the *X*-axis by the magnetometer is not a true horizontal *X*-axis component. Therefore, the attitude matrix is introduced to deal with this problem.

The correspondences of the magnetometer triaxial output at two adjacent moments can be expressed as:

(7) $$C_{1}X_{1}=C_{2}X_{2}$$

$C_{1}$ and $C_{2}$ are the attitude matrices corresponding to the two moments of the magnetometer, respectively. $X_{1}$ and $X_{2}$ are the triaxial output vectors of the magnetometers at two moments. The triaxial correspondence described above can be expressed as:

(8) $$C_{b}^{n}\left(k\right){\hat{M}}^{b}\left(k\right)=C_{b}^{n}\left(k+1\right){\hat{M}}^{b}\left(k+1\right)$$

$C_{b}^{n}\left(k\right)$ is the direction cosine matrix of the platform from coordinate system (b) to the navigation coordinate system (n) at time of k. $C_{b}^{n}\left(k+1\right)$ is the direction cosine matrix at time of k+1. ${\hat{M}}^{b}\left(k\right)$ is the triaxial output vector of the corrected magnetometer at time of k. ${\hat{M}}^{b}\left(k+1\right)$ is the vector at time of k+1.

The geographic coordinate system Northeastern (ENU) is selected as the navigation coordinate system (n-system); then, the cosine matrix of the platform from b-system to the n-system is:

(9) $$C_{b}^{n}=[\begin{array}{ccc} sin\gamma cos\psi -sin\gamma sin\theta sin\psi & -cos\theta sin\psi & -sin\gamma cos\psi +sin\theta cos\gamma sin\psi \\ cos\gamma sin\psi +sin\gamma sin\theta cos\psi & cos\theta cos\psi & sin\gamma sin\psi -sin\theta cos\gamma cos\psi \\ -sin\gamma cos\theta & sin\theta & cos\gamma cos\theta \end{array}]$$

where ψ is yaw angle. θ is pitch angle. γ is roll angle. θ and γ can be calculated by the follows:

(10) $$\theta =-{tan}^{-1}\frac{A_{y}}{\sqrt{A_{x}^{2}+A_{y}^{2}+A_{z}^{2}}}$$

(11) $$\gamma ={tan}^{-1}\frac{A_{x}}{\sqrt{A_{x}^{2}+A_{y}^{2}+A_{z}^{2}}}$$

$A_{x}$, $A_{y}$ and $A_{z}$ are the output values of the triaxial accelerometer, respectively.

Since each coordinate maintains a Cartesian coordinate system in the equivalent rotation of the b-to-n-system, $C_{b}^{n}$ is a unit orthogonal matrix. Then, $C_{b}^{n}={\left(C_{n}^{b}\right)}^{-1}={\left(C_{n}^{b}\right)}^{T}$. According to Equation (8),

(12) $$\begin{array}{cc} {\hat{M}}^{b}\left(k+1\right) & ={\left(C_{b}^{n}\left(k+1\right)\right)}^{-1}C_{b}^{n}\left(k\right){\hat{M}}^{b}\left(k\right)\\ & ={\left(C_{b}^{n}\left(k+1\right)\right)}^{T}C_{b}^{n}\left(k\right){\hat{M}}^{b}\left(k\right) \end{array}$$

It also can be obtained from Equation (6) that

(13) $$\begin{array}{cc} {\hat{M}}^{b}\left(k+1\right) & =N\left(M^{h}\left(k+1\right)-E\left(K+1\right)\right)\\ & =NM^{h}\left(k+1\right)-NE\left(K+1\right) \end{array}$$

Therefore,

(14) $${\left(C_{b}^{n}\left(k+1\right)\right)}^{T}C_{b}^{n}\left(k\right){\hat{M}}^{b}\left(k\right)=NM^{h}\left(k+1\right)-NE\left(K+1\right)$$

Letting $C_{b}^{n}\left(k\right){\hat{M}}^{b}\left(k\right)={\left(\begin{array}{ccc} x_{1} & y_{1} & z_{1} \end{array}\right)}^{T}$, $NM^{h}\left(k+1\right)={\left(\begin{array}{ccc} x_{2} & y_{2} & z_{2} \end{array}\right)}^{T}$, and $NE\left(K+1\right)={\left(\begin{array}{ccc} n_{1} & n_{2} & n_{3} \end{array}\right)}^{T}$, the following can be obtained:

(15) $$\left[\begin{array}{c} x_{2}-n_{1}\\ y_{2}-n_{2}\\ z_{2}-n_{3} \end{array}\right]=\left[\begin{array}{ccc} sin\gamma cos\psi -sin\gamma sin\theta sin\psi & cos\gamma sin\psi +sin\gamma sin\theta cos\psi & -sin\gamma cos\theta \\ -cos\theta sin\psi & cos\theta cos\psi & sin\theta \\ -sin\gamma cos\psi +sin\theta cos\gamma sin\psi & sin\gamma sin\psi -sin\theta cos\gamma cos\psi & cos\gamma cos\theta \end{array}\right]\left[\begin{array}{c} x_{1}\\ y_{1}\\ z_{1} \end{array}\right]$$

(16) $$\;\begin{array}{l} =[\begin{array}{c} \left(sin\gamma cos\psi -sin\gamma sin\theta sin\psi \right)x_{1}+\left(cos\gamma sin\psi +sin\gamma sin\theta cos\psi \right)y_{1}-\left(sin\gamma cos\theta \right)z_{1}\\ -\left(cos\theta sin\psi \right)x_{1}+\left(cos\theta cos\psi \right)y_{1}+\left(sin\theta \right)z_{1}\\ \left(-sin\gamma cos\psi +sin\theta cos\gamma sin\psi \right)x_{1}+\left(sin\gamma sin\psi -sin\theta cos\gamma cos\psi \right)y_{1}+\left(cos\gamma cos\theta \right)z_{1} \end{array}]\\ =[\begin{array}{c} \left(x_{1}sin\gamma +y_{1}sin\gamma sin\theta \right)cos\psi +\left(y_{1}cos\gamma -x_{1}sin\gamma sin\theta \right)sin\psi -\left(sin\gamma cos\theta \right)z_{1}\\ \left(y_{1}cos\theta \right)cos\psi -\left(x_{1}cos\theta \right)sin\psi +\left(sin\theta \right)z_{1}\\ \left(-x_{1}sin\gamma -y_{1}sin\theta cos\gamma \right)cos\psi +\left(x_{1}sin\theta cos\gamma +y_{1}sin\gamma \right)sin\psi +\left(cos\gamma cos\theta \right)z_{1} \end{array}] \end{array}$$

where ${\left(\begin{array}{ccc} x_{1} & y_{1} & z_{1} \end{array}\right)}^{T}$ are the triaxial output of the magnetometer at time *k*, ${\left(\begin{array}{ccc} x_{2} & y_{2} & z_{2} \end{array}\right)}^{T}$ are the output of the magnetometer at time k+1, and ${\left(\begin{array}{ccc} n_{1} & n_{1} & n_{1} \end{array}\right)}^{T}$ represents the interference on three axes. Both γ and θ are known. The unknown variable are $\left(\begin{array}{cc} \begin{array}{cc} \psi & n_{1} \end{array} & \begin{array}{cc} n_{2} & n_{3} \end{array} \end{array}\right)$

According to the idea of the limit, when the sampling time is short enough, the strength of the surrounding magnetic fields at the two adjacent moments is equal. It has the following relationship: $\left\|M_{k}\|=\|M_{k+1}\right\|$; then, ${\left\|N\left(M^{h}-E\right)\right\|}_{k}={\left\|N\left(M^{h}-E\right)\right\|}_{k+1}$. Since the value at time k is known, letting $M_{k}=M_{0}$,

(17) $$\|\begin{array}{c} x_{2}-n_{1}\\ y_{2}-n_{2}\\ z_{2}-n_{3} \end{array}\|=\|\begin{array}{c} x_{1}\\ y_{1}\\ z_{1} \end{array}\|=M_{0}$$

(18) $${\left(x_{2}-n_{1}\right)}^{2}+{\left(y_{2}-n_{2}\right)}^{2}+{\left(z_{2}-n_{3}\right)}^{2}=M_{0}^{2}$$

According to Equations (16) and (18) and ${\left(\sin\psi \right)}^{2}+{\left(\cos\psi \right)}^{2}=1$, ψ can be obtained.

### 3.2. GNSS/INS Integration Based Kalman Filter

**Figure 4:**
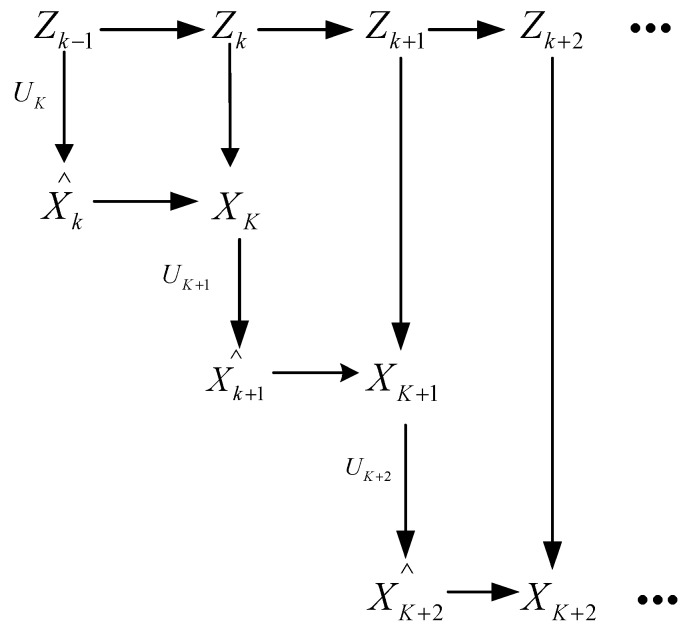
The process of solving the optimal value.

To reduce the noise in the GNSS signal, a basic Kalman filter is selected in this paper. We take the measurement result of the GNSS receiver as the observation value, and the measurement data of the IMU as the increment, and the predicted value is obtained by these two values, as shown in Figure 4. The observation result of GNSS at *k* − 1 time is $Z_{k-1}$ and $U_{k}$ is the position increment obtained by IMU data at *k* time. The *k* time predicted value ${\hat{X}}_{k}$ is obtained by Equations (2) and (3). Then, according to ${\hat{X}}_{k}$ and $Z_{k}$, the Kalman filter is used to solve the optimal value $X_{k}$ at time *k*. At the next moment, the *k* time optimal value $X_{k}$ and the $U_{k+1}$ are used to obtain the predicted value ${\hat{X}}_{k+1}$ at *k* + 1 time. Then, with $Z_{k+1}$, the *k* + 1 time optimal value $X_{k+1}$ is obtained by Kalman filter, and so on. The sampling time of the system is set to 0.1 s. In addition, the magnetometer provides the initial direction for data conversion of the INS.

The formulation is described as follows:

(19) $${\hat{X}}_{k}=\phi \times X_{k-1}+U_{k}$$

(20) $${\hat{p}}_{k}=\phi \times P_{k-1}\times \phi^{T}+Q_{k}$$

(21) $$K={\hat{p}}_{k}\times H_{k}^{T}\times {\left(H_{k}\times {\hat{p}}_{k}\times H_{k}^{T}+R_{k}\right)}^{-1}$$

(22) $$X_{k}={\hat{X}}_{k}+K_{k}\times \left(Z_{k}-H_{k}\times {\hat{X}}_{k}\right)$$

(23) $$P_{k}=\left[I-K_{k}\times H_{k}\right]\times {\hat{p}}_{k}$$

where the subscript denotes the *k*th epoch, and the caret ∧ indicates a Kalman filter estimate. X is the state vector of longitude (Lng) and latitude (Lat) as follows:

(24) $$X=\left[\begin{array}{c} Lng\\ Lat \end{array}\right]$$

U is the system input provided by the INS which includes acceleration a and angular velocity ω. The changes in position (ΔLng and ΔLat) are obtained by the following formulas:

(25) $$U=\left[\begin{array}{c} \Delta Lng\\ \Delta Lat \end{array}\right]$$

(26) $$\Delta Lng=\left[v_{k}T_{0}+\frac{1}{2}T_{0}^{2}a_{k}\right]cos(\alpha_{k-1}+\omega_{k}T_{0})B$$

(27) $$\Delta Lat=\left[v_{k}T_{0}+\frac{1}{2}T_{0}^{2}a_{k}\right]sin(\alpha_{k-1}+\omega_{k}T_{0})A$$

Z is the measurement provided by the commercial GNSS receiver as follows:

(28) $$Z=\left[\begin{array}{c} Lng_{gnss}\\ Lat_{gnss} \end{array}\right]$$

H is the observation matrix and ϕ is the system propagation matrix. Both H and ϕ are used as a unity matrix in this integration. P is the state covariance. *K* is the Kalman gain. The process noise covariance (Q) and the measured noise covariance (R) affect the Kalman gain, which is the weight between the system prediction and the measurement update [24,25]. In general, Q and R are fixed values, resulting in a constant weighting between GNSS and INS. However, the environment between cities is different. Subsequently, constant tuning cannot yield an optimal performance [26]. An adaptive tuning algorithm is needed to describe the noise of the GNSS measurement model. An adaptive Kalman filter based on GNSS/INS integration scheme specifically was proposed for commercial flight control system [26]. This article assigns values to R and Q based on its classification results. 

## 4. Experiment

The system platform consists of a processor unit, a GNSS receiver, an IMU and a magnetometer. The processor unit is mainly composed of OMAPL138 processor and its peripherals. It has an ARM plus DSP dual core architecture. ARM kernel of OMAPL138 processor runs Linux operation system responsible for control of the entire system and data acquisition. DSP kernel is responsible for the data calculation. The NV08C-CSM is a fully integrated multi-constellation satellite navigation receiver which can offer high precision and low power consumption. The NV08C-CSM supports National Marine Electronics Association (NMEA) protocol and binary (BINR) protocol. Compared with NMEA protocol which is a unified Radio Technical Commission for Maritime standard in different GNSS navigation devices, BINR protocol can receive more comprehensive satellite navigation raw data such as pseudo-range and signal-to-noise ratio to meet more navigation algorithms. The IMU chip can measure triaxial accelerations and triaxial angular velocity of the platform. The magnetometer chip can also measure triaxial magnetic field strength. 

**Figure 5:**
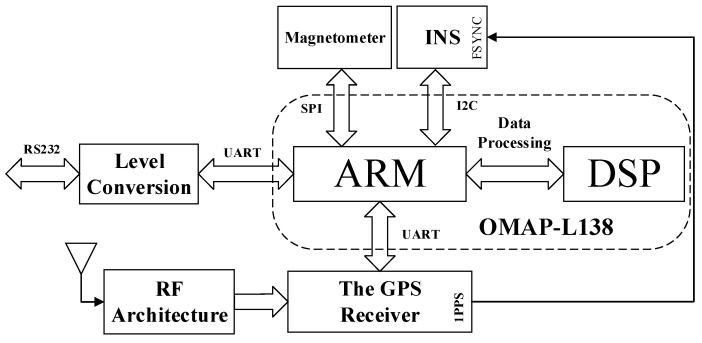
The architecture of the proposed system platform.

The data received by the processor module not only come from the GNSS receiver NV08C-CSM, but also the IMU and magnetometer. It is shown in Figure 5. During this process, the embedded processor sends BINR request command to the NV08C-CSM. The module receives the satellite signal through the antenna, and sends the BINR response message back to the processor through the Universal Asynchronous Receiver/Transmitter (UART). The IMU sends the data of triaxial acceleration and triaxial angular velocity of the platform to the processor by Inter-Integrated Circuit (I2C). The magnetometer sends the triaxial magnetic field strength to the processor by Serial Peripheral Interface (SPI). The above acquisition process is treated by the ARM, and then the data are sent to the DSP to be decoded, filtered, and fusion. Finally, the processed data are returned to the ARM and sent out through the UART. The TTL level is converted to the RS232 format output through the MAX3232 chip.

**Figure 6:**
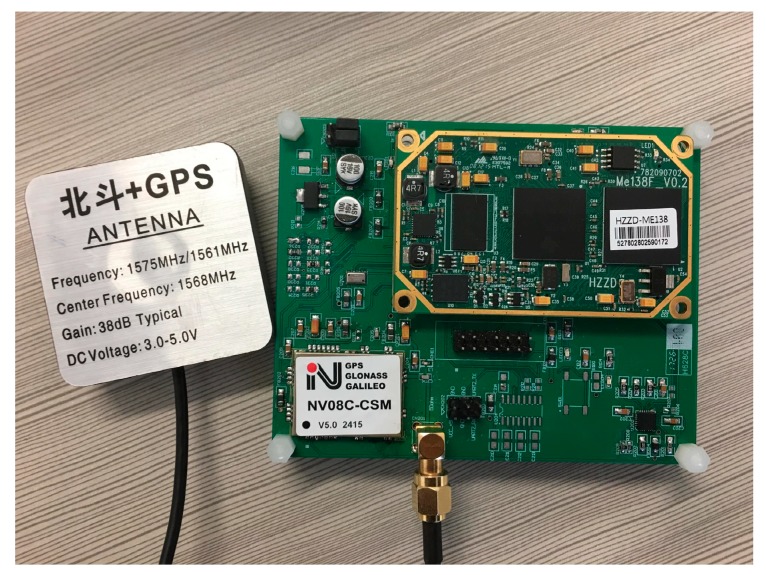
Hardware platform.

In view of the design described above, we tested every unit to ensure the circuit was correct after finishing the hardware platform. The hardware platform is shown in Figure 6. Then, the Linux operating system was transplanted on this platform.

After completing the data acquisition, the system needs to transfer the data from the ARM to the DSP. In this study, the SYSTEM LINK (SYSLINK) provided by Texas Instruments was used to achieve ARM and DSP dual-core communication. It provides a way to connect software across multiple cores with each processor running an operating system such as Linux, Quick Unix, etc. Then, it transports the data to the DSP through the SYSLINK. DSP kernel is used for the data filtering and navigation algorithm.

**Figure 7:**
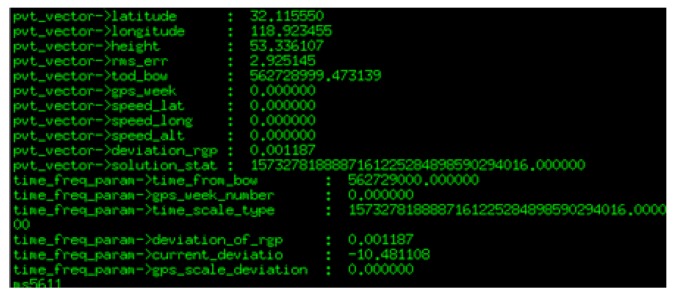
Partial measurement data.

Before testing the performance of the platform, it some preparation and initial calibration were needed. First, the platform was connected to a computer to check whether its data were normal. The serial port settings were as follows: 115,200 bit/s baud rate, 8 data bits, 1 stop bit and no parity. Then, we started to test the performance of the board after the data acquisition program ran. The display data on the terminal are shown in Figure 7.

Usually, the magnetic field sensor has a large zero error in manufacturing. If it is not calibrated, it will bring a large measurement error. The magnetic field calibration is used to remove the zero bias of the magnetic field sensor.

**Figure 8:**
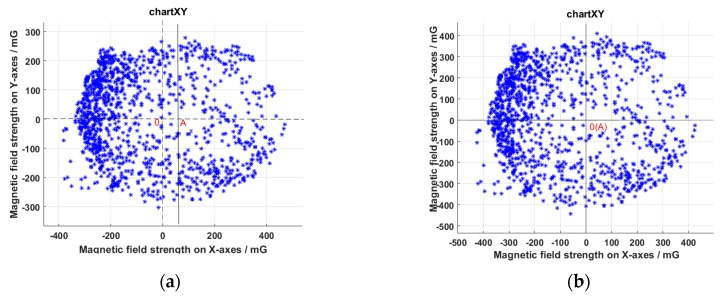
Magnetic field projected on the XY plane: (**a**) uncorrected data; and (**b**) corrected data.

The magnetometer in the platform could measures the magnetic field strength of three axes. The space vector composed of triaxial component is actually the position vector of the Earth’s magnetic field relative to the sensor. The direction of the sensor is constantly changing during the platform movement. The data are output in order while the magnetometer reads the three-axis magnetic field. We rotated the sensor around each axis so that a graph consisting of the endpoints of the vectors at each moment should be a sphere, which is centered on the origin. Figure 8a is a view projected on the XY plane. The data on the *Y*-axis are distributed on both sides of Point 0, which is symmetrical. However, the center Point A on the *X*-axis deviated from Point 0. Since the data measured by the sensor in both the positive and negative directions of the magnetic field should be the same value but opposite sign, i.e. it should be a circle centered on the origin, this situation showed that the data had zero drift.

The correction method was to sum the maximum and minimum values of the *X*-axis, and then half of the sum was the zero offset compensation value of the *X*-axis data. The corrected image is shown in Figure 8b. It is a circle centered on Point 0. Point A coincides with Point 0 at this moment. Similar processing was done on the data on the *Z*-axis. In this way, the angle between the heading of the carrier and the magnetic north could be obtained.

**Figure 9:**
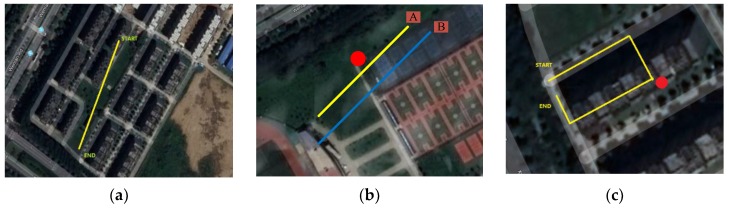
Testing scenarios for the proposed integrated positioning system: (**a**) testing the positioning effect of GNSS/INS; (**b**) testing the correction effect under the interference of different magnetic field strength; and (**c**) testing platform seamless positioning performance.

As shown in Figure 9, three scenarios were tested, as detailed in Section 4.1, Section 4.2 and Section 4.3, respectively. In this study, the true trajectories were obtained by multiple measurements using high-precision aerial mapping vehicles.

### 4.1. Experiment Result for GNSS/INS Filtering

**Figure 10:**
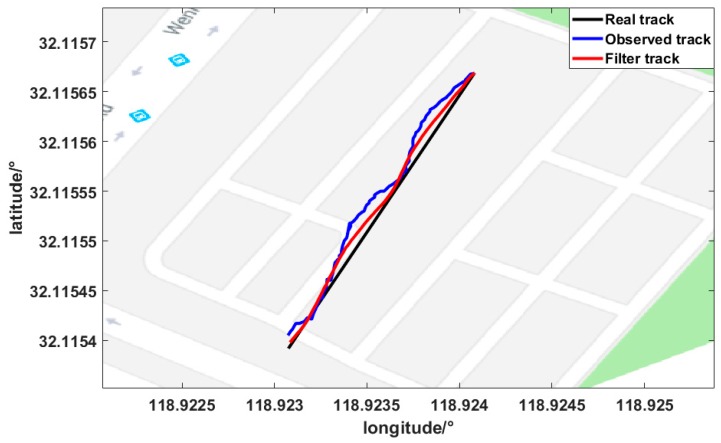
Track of movement.

**Figure 11:**
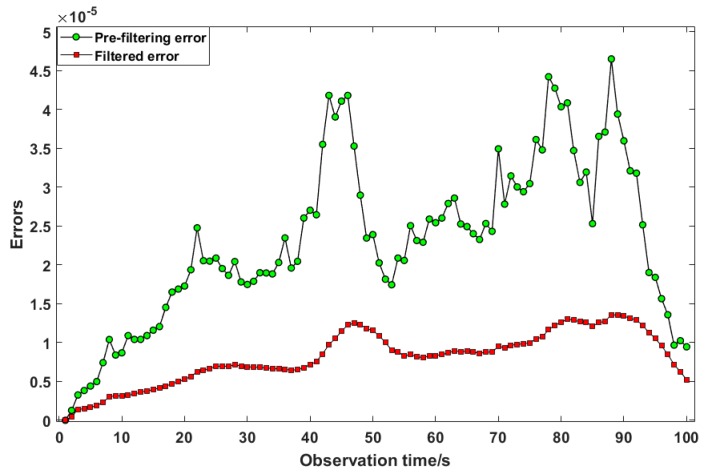
Tracking error.

The outdoor experiment was to test the outdoor positioning performance of the mobile platform, as shown in Figure 9a. The track started at START point and ended at END point. The buildings were distributed along two sides of the track. The output positioning of the mobile platform is shown in Figure 10. The black line indicates the true trajectory, the blue line indicates the trajectory of the GNSS receiver output, and the red line indicates the trajectory after filtering. As can be seen in the figure, the blue line has a more obvious oscillation. After filtering, the trajectory oscillated less, which was closer to the real trajectory. In Figure 11, the red line represents the error between the filtered trajectory and the real trajectory, while the green line represents the error between the GNSS observation trajectory and the real trajectory. Compared with the green line, the error was reduced by approximately 60%. The mean and standard deviation (STD) of the positioning errors are summarized in Table 1. The formula for the mean of positioning error is as follows:

(29) $$Mean=\frac{error_{1}+error_{2}+\cdots +error_{n}}{n}$$

The formula for STD of positioning error is as follows:

(30) $$STD=\sqrt{\frac{1}{n}\overset{n}{\underset{i=1}{\sum }}{\left(Mean-error_{i}\right)}^{2}}$$

The error mean and STD of uncorrected positioning were 1.6025 m and 0.7 m, respectively. The corrected positioning achieved a performance of 0.7 m and 0.22 m in the mean and STD of the error, respectively. The data also reflect an accuracy of about 60%.

### 4.2. Experiment Result for Heading Angle Correction of Magnetometer

**Figure 12:**
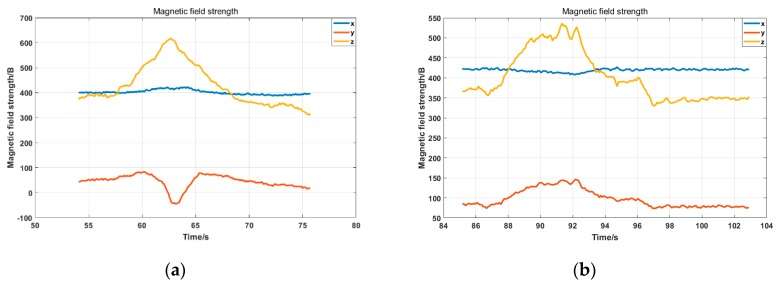
Output of triaxial magnetometer: (**a**) triaxial magnetic field data under large interference; and (**b**) triaxial magnetic field data under small interference.

**Figure 13:**
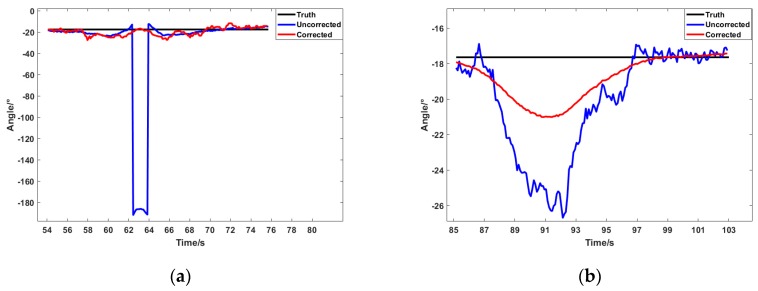
Heading angle results for two paths: (**a**) angle in large interference; and (**b**) angle in small interference.

The indoor experiment for testing the proposed mobile platform was conducted beside a source of magnetic interference, as shown in Figure 9b. The red dot in the figure indicates the location of the interference source. A magnet was used as the interference source. Paths A and B were, respectively, placed next to the interference source. The distance between Path A and the interference source was set to 0.1 m, and the distance set between Path B and the interference source was 0.6 m. This paper proposes a method that calculates and corrects the heading angle dynamically, and the purpose of the experiment was to test the effect of method under different interference intensities. The data of triaxial magnetometer under Paths A and B are shown in Figure 12a,b, respectively. The calculated heading angle is shown in Figure 13. Since both Paths A and B were straight trajectories without angular change, the value of the black line in the figure is unchanged, indicating the real angle.

The blue line indicates the uncorrected angle. Since it was closer to the interference source, the value is severely affected and the wrong part can be is seen in Figure 13a. The red line indicates the angle after correction. In Figure 13a, it does not have the same serious deviation as the blue line, and it restores the true angle more closely. The mean and STD values of the angle errors are summarized in Table 2. The error mean and STD of uncorrected angle were 13.4502 ° and 42.9705 °, respectively. The corrected angle achieved a performance of 2.1278 ° and 3.7276 ° in the mean and STD of the error, respectively. The data also reflect an accuracy of about 60%. The true angle was greatly restored.

Figure 13b shows the case of Path B. Since Path B was slightly farther from the source of interference, its value was not as severely affected. The maximum error between the blue line and the black line is about 8 °. After the correction, the error was reduced to three °. The mean and STD of the angle errors are summarized in Table 3. The error mean and STD of uncorrected angle were 2.467 °and 2.9812 °, respectively. The corrected angle achieved a performance of 1.2207 ° and 1.2267 ° in the mean and STD of the error, respectively. The accuracy was increased by about 50%.

In Figure 13a, the corrected line (red) points towards the truth while the red line in Figure 13b maintains a similar shape to the uncorrected line. The reason can be understood in conjunction with Equations (17) and (18). When the sampling time is short enough, the magnetic field strength at time *k* is the same as the time *k* + 1. When a part of the interference is filtered as the parameter n, the direction of the *k* + 1 moment should be maintained at the previous moment. Therefore, the corrected line (red) points towards the truth in Figure 13b. There are two differences between Figure 13a,b: the intensity of the interference and the disturbed time. As shown in Figure 13a, the interfering time was 62 s to 64 s, indicating that it was moving more quickly. The data are abrupt at 62 s, and the value at 63 s should be kept at 62 s according to the above. Thus, it pointed towards the truth.

### 4.3. Experiment Result for the Performance of Platform Seamless Positioning

**Figure 14:**
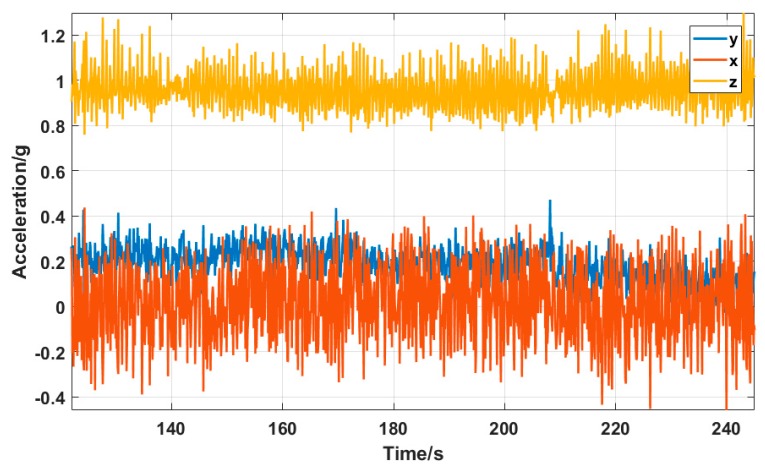
Triaxial acceleration data.

**Figure 15:**
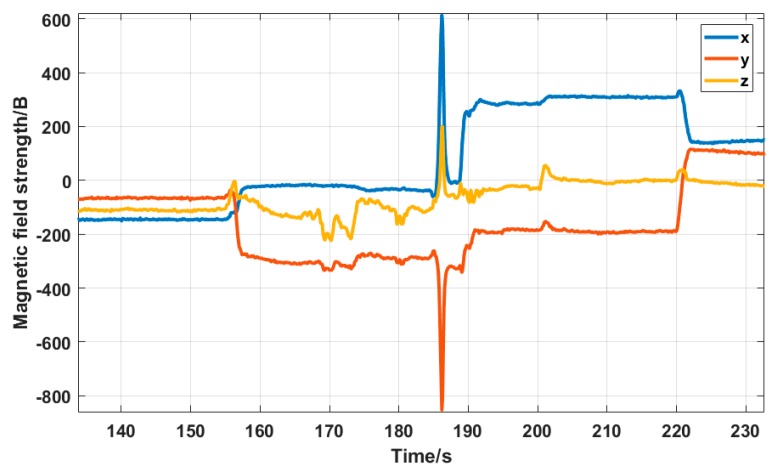
Triaxial magnetic field strength.

The third experiment for testing the proposed mobile platform was a path passing through both indoor and outdoor environment. As shown in Figure 9c, the lab building and the surrounding area were chosen to test the performance of the positioning system. The entire path covered both outdoor and indoor environment, and three turns were set. A magnetic field interference source was added at the second turn to test the positioning performance. The outdoor part of the path adopted the method depicted in Section 4.1, which uses GNSS and INS data in combination with Kalman filtering. The indoor part used the method in Section 4.2, and the trajectory estimation was performed based on the heading angle solved by the magnetometer. Figure 14 shows the data read by the triaxial accelerometer during platform motion. The unit of the vertical axis is *g*, i.e. the gravity acceleration of the Earth. The yellow line indicates the data in the *Z*-axis direction, which points to the ground, thus the value is about 1× *g*. Since the platform moved along the *Y*-axis, the direction had acceleration. It can be seen in the figure that the data on the *Y*-axis are not 0. There was no force on the *X*-axis, so its value is 0. Figure 15 shows the data of the triaxial magnetometer during the platform motion. 

**Figure 16:**
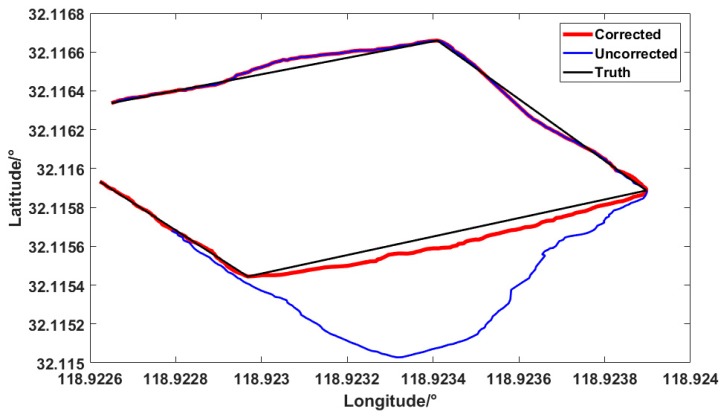
Track of seamless the indoor/outdoor movement.

The data output of the platform were imported into Google Maps, as shown in Figure 16. The red line in the figure represents the true trajectory, and the blue line represents the positioning of the platform output. In general, the blue line restores the real path approximately. The mean and STD of the positioning errors are summarized in Table 4. The error mean and STD of corrected positioning were 1.73 m and 1.44 m, respectively. The error of overall positioning trajectory and true trajectory was within 2 m. The result verifies the proposed method could meet the civil accuracy requirements in general operations.

In Figure 16, since the magnetometer is disturbed at the second corner, and the uncorrected heading angle is deviated, causing the trajectory to deviate from the original trajectory. Thus, the blue line deviates from the black line after the turning. After error correction, the affected degree of trajectory is greatly reduced. Therefore, the red line surrounds the real trajectory.

### 4.4. Discussions

The method of calculating heading angle proposed in this paper is based on magnetometer sensing data. In other methods, the heading angle can be directly obtained based on the current reading of the magnetometer. Different from that, the principle of the propose method is to separate the value n of interference and the true value of the magnetic field strength in the working environment. The value n and the value of heading angle can be obtained using Equation (15). The method achieved better performance when interference existed. It needs to be pointed out that the method is assumes that the magnetic field strength remains unchanged in the continuous two sampling time slots. To achieve that, the sampling time of magnetometer sensors must be set fast enough. For example, if the sampling time of the magnetometer is set to 6 s, the interference in the time slot between 62 s and 64 s in Figure 13a cannot be observed. Correspondingly, the method cannot get the correct solution if the head angle changes during the time. It also can be concluded by Equation (15) that the heading angel cannot be correct if interference exists in time zero.

## 5. Conclusions

In this paper, an embedded integrated positioning platform is presented. The platform was designed to acquire the data of GNSS, magnetometer and INS for positioning in seamless indoor/outdoor scenes. In this work, three main points are considered: First, a method to dynamically calculate and correct the heading angle is proposed, which improves the accuracy of positioning by reducing interference. Secondly, to reduce the noise in the GNSS signal, the GNSS/INS combination method by Kalman filtering is used. Finally, ARM+DSP structure is adopted to implement the functionalities of system control and calculation. Experiments were developed along planned paths on multiple occasions. Experimental results show that the method of heading angle correction could improve the accuracy by at least 60%. The error of overall positioning trajectory and true trajectory was within 2 m. In addition, the platform ran the multi-channel acquisition program steadily during the movement. It showed that the system had good performance. Furthermore, some of the platform needs to be improved. When the board is running, some chips will heat, which affects the performance of the system. Therefore, they should be separated by a little distance between in the future design. Since GNSS receiver can also receive some raw data such as pseudo-range, carrier phase, etc., these data can be used to do some tight GNSS/INS integration in the future.

## Figures and Tables

**Table 1:** Test Occasion 1: Mean and STD of the positioning error for the GNSS Observation and Kalman Filter.

	GNSS Observation	Kalman Filter
Mean of positioning error (m)	1.6025	0.5626
STD of positioning error (m)	0.7	0.22

**Table 2:** Test Occasion 2: Mean and STD of the heading angle error under large interference.

	Uncorrected	Corrected
Mean of angle error (°)	13.4502	2.1278
STD of angle error (°)	42.9705	3.7276

**Table 3:** Test Occasion 2: Mean and STD of the heading angle error under small interference.

	Uncorrected	Corrected
Mean of angle error (°)	2.4670	1.2207
STD of angle error (°)	2.9812	1.2267

**Table 4:** Test Occasion 4: Mean and STD of the positioning error of the seamless indoor/outdoor positioning.

	Uncorrected	Corrected
Mean of positioning error (m)	8.45	1.73
STD of positioning error (m)	10.77	1.44
